# In Vivo MR Microneurography of the Tibial and Common Peroneal Nerves

**DOI:** 10.1155/2014/780964

**Published:** 2014-12-07

**Authors:** Paolo F. Felisaz, Eric Y. Chang, Irene Carne, Stefano Montagna, Francesco Balducci, Giulia Maugeri, Anna Pichiecchio, Fabrizio Calliada, Maurizia Baldi, Stefano Bastianello

**Affiliations:** ^1^Radiology Department, University of Pavia, 27100 Pavia, Italy; ^2^Radiology Service, VA San Diego Healthcare System, San Diego, CA 92161, USA; ^3^Medical Physics Department, IRCCS Salvatore Maugeri Foundation, Scientific Institute of Pavia, 27100 Pavia, Italy; ^4^Radiology Department, IRCCS Salvatore Maugeri Foundation, Scientific Institute of Pavia, 27100 Pavia, Italy; ^5^Neuroradiology Department, C. Mondino National Neurological Institute, 27100 Pavia, Italy; ^6^Institute of Radiology, IRCCS Policlinico S. Matteo Foundation, 27100 Pavia, Italy; ^7^Department of Brain and Behavioral Sciences, University of Pavia, 27100 Pavia, Italy

## Abstract

MR microneurography is a noninvasive technique that provides visualization of the microanatomy of peripheral nerves, otherwise available only with histopathology. The objective of this study was to present a protocol to visualize the microstructure of peripheral nerves in vivo, using a 3T MRI scanner with a clinical set of coils and sequences. The tibial and the common peroneal nerves of healthy volunteers were imaged above the medial malleolus and at the level of the fibular head, respectively. The acquired images provided details about the internal structure of peripheral nerves, with visualization of the fascicles, the interfascicular fat, the epineurium, and the perineurium. MR microneurography can be performed in a clinical setting with acceptable imaging times and can be a potentially powerful tool that complements standard MR neurography.

## 1. Introduction

Magnetic resonance imaging (MRI) of peripheral nerves, also known as MR neurography, provides visualization of the main peripheral nerve trunks and allows detection of pathologic changes such as edema, loss of fascicular pattern, osteofibrous tunnel narrowing, and tumors [1]. Functional evaluation with diffusion weighted imaging (DWI) and diffusion tensor imaging (DTI) techniques have also been described [2]. One main issue with standard MR neurography is spatial resolution, in fact, the nerve fibers, the fascicles and the connective tissues within the peripheral nerves are not easily accessible with conventional imaging protocols.

However, the visualization of the microanatomy of peripheral nerves is a matter of clinical relevance. According to the commonly utilized Seddon and Sunderland classifications for peripheral nerve injuries [3, 4], the integrity of the connective tissues such as the epineurium and perineurium is related to clinical outcome [5]. Current standard of care utilizes a combination of clinical findings and electrophysiology for diagnosis. However, a technique that can visualize and evaluate earlier the integrity of the epineurium and the perineurium may potentially improve diagnosis.

MR microneurography is a noninvasive technique that provides visualization of anatomic details otherwise available only with histopathology. Since early ex vivo experiments that were performed with high field scanners, there has been little development in this field [5–7]. The objective of this study was to present a protocol of MR microneurography using a 3T scanner and a clinical set of coils and sequences, which could potentially visualize the microarchitecture of peripheral nerves in vivo.

## 2. Materials and Methods

Imaging was performed on a Discovery MR750 3T scanner (GE Healthcare, Milwaukee) utilizing a 6-channel carotid array coil. Each antenna set articulates, rotates, and locks facilitating the setup in the ankle and knee regions.

After acquisition of written, informed consent, imaging was performed on five volunteers (24 to 30 years of age), all referred in good health without clinical complaints. Experiments were repeated several times with different standard coils and sequences with the aim of finding the optimal setup. Only a few published protocols are available in the literature [5–10] and, to the best of our knowledge, there are no published standard protocols for use on clinical scanners. The 6-channel carotid coil was utilized because it provided high signal-to-noise-ratio (SNR) for relatively superficial nerves. This coil allowed comfortable positioning on the lower limb for the study of the ankle and the knee regions. The tibial nerve was imaged several centimeters along its course, from above the tibiotalar joint into the tarsal tunnel. The common peroneal nerve (CPN) was imaged several centimeters along its course, from above the fibular head to the region of the fibular neck.

3D spoiled gradient echo technique (SPGR—GE, FLASH—Siemens, T1 FFE—Philips) was chosen because it has the advantages of 3D acquisition, with preservation of SNR and lower SAR compared with 3D fast-spin-echo (FSE) techniques such as SPACE (Siemens), CUBE (GE), or VISTA (Philips). Additionally, this particular class of sequences is available on nearly all 1.5T and 3T scanners, which is not the case with the 3D FSE sequences. The detailed parameters of the sequences used are reported in Table 1.

Low-resolution images were first acquired using 3D SPGR sequence with FOV of 10–14 cm. This was useful to localize the neurovascular bundle and visualize the nerve course, facilitating the selection of an exactly perpendicular plane to the nerve, a crucial point to counteract partial volume effects (see Section 4.2).

Thereafter, high-resolution images were acquired with smaller FOVs, ranging from 3 to 6 cm, 512 × 480 matrix, with scan time approximately 10–12 minutes per sequence. All sequences were acquired without contrast agent injection. Fluid sensitive images were obtained using 3D SPGR sequences with a small flip angle (10°), adding a standard fat saturation preparation pulse. T1 weighted images were obtained using 2D fast spin echo sequences.

## 3. Results

**Figure 1:**
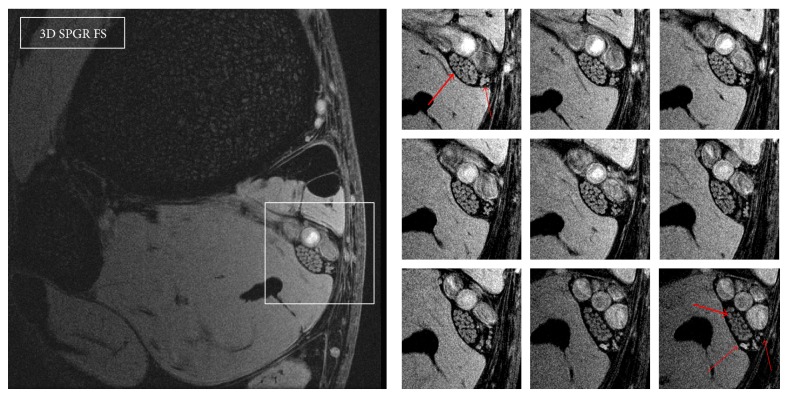
3D SPGR FS, FOV 5 cm. Axial sections at the posteromedial aspect of the tibia, above the medial malleolus. The tibial nerve is visualized (large arrow) with the typical fascicular pattern. The neurovascular bundle is seen at the posteromedial aspect of the tibia. Sequential images at high resolution demonstrate the medial calcaneal nerve dividing into anterior and posterior branches, providing sensory innervation to the plantar aspect of the heel (small arrows).

**Figure 2:**
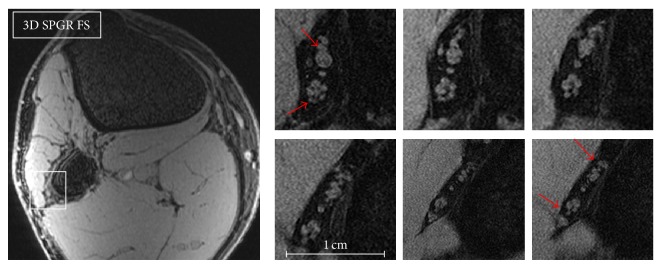
3D SPGR FS, FOV 5 cm. Axial sections of the common peroneal nerve (CPN) at the level of the fibular neck. The CPN is approximately half the diameter of the tibial nerve and therefore contains fewer fascicles. It travels along the lateral aspect of the fibular neck and divides into two main branches, the deep and superficial peroneal nerves (arrows).

**Figure 3:**
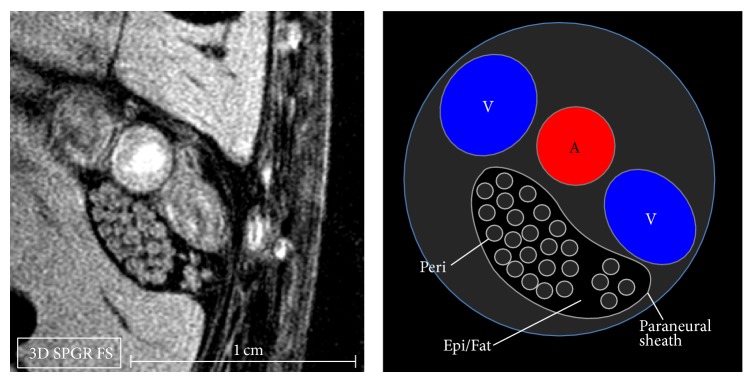
3D SPGR FS. Axial section of the neurovascular bundle at the ankle with the tibial nerve and the corresponding schematic diagram. In-plane resolution is ~100 *μ*m. The paraneural sheath is hyperintense. The epineurium and the epineurial fat (Epi/Fat) appear hypointense because the fat is suppressed and the epineurium has a fibrous structure with low signal. The perineurium (Peri) is hyperintense. The artery (A) demonstrates a thicker wall compared with the paired veins (V). The neurovascular bundle is surrounded by a fatty, supporting tissue and enclosed within a thicker connective fascia.

**Figure 4:**
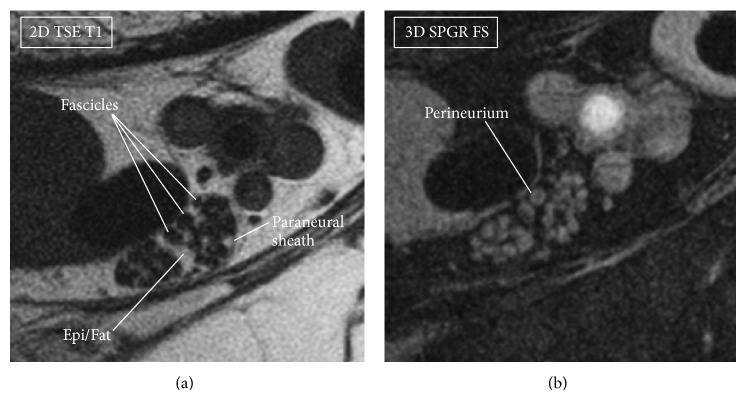
Tibial nerve, axial sections. In 2D TSE T1 weighted image (a) the fascicles are hypointense surrounded by a hyperintense tissue, mainly the epineurial fat (Epi/Fat), while the fibrous part of the epineurium has low signal and surrounds the fascicles; it is not clearly seen. The paraneural sheath is also detected. In SPGR fat suppressed image (b), the fascicles are covered by a bright thin layer, the perineurium, best seen when the acquisition plane is exactly perpendicular to the nerve orientation, therefore minimizing the partial volume effects.

Typical microneurograms of the tibial nerve at the ankle and the CPN at the fibular head-neck, using the above MRI protocols, are shown in Figures 1–4. Images of these two nerves were obtained from all the volunteers, but best results were possible when the acquisition plane was precisely orthogonal to the long axis of the nerve, thereby minimizing partial volume effects. Anatomical nerve course variations within the acquisition coverage (2 cm) such as curving, bending, or divisions were a limit to spatial resolution. The localizer protocol (3D SPGR, low resolution with large coverage) was critical to select straight nerve tracts. The tibial nerve was easier to study since its diameter is approximately twice of the CPN, and it exhibited usually a straight course above the tibial malleolus. The CPN instead travels in a curved tract before the fibular head and it divides into its two deep and superficial branches in the peroneal tunnel; therefore imaging of the CPN was more difficult.

On the images in Figures 1 and 2, sequential axial sections of the tibial nerve and the CPN demonstrate the typical fascicular pattern. Details on the neurovascular bundle at the ankle and the main branches of these two nerves are pointed out in the figures.  Figure 3 shows the constituents of the neurovascular bundle at the ankle, composed of the tibial nerve, posterior tibial artery, and two veins, surrounded by a fatty, supporting tissue and enclosed within a thicker connective fascia. The tibial nerve is seen with approximately 100 *μ*m in-plane resolution. On 3D SPGR fat suppressed images, the fascicles are hyperintense and highlighted against a hypointense background, mainly due to the suppressed epineurial fat and the epineurial fibrous tissue. This becomes clear in the T1 weighted image (Figure 4), where the epineurial fat is hyperintense and the fascicles are hypointense. The fibrous part of the epineurium is hypointense and surrounds the fascicles, which is the reason why it is not clearly visible. The perineurium is hyperintense in the 3D SPGR fat suppressed images and is best demonstrated when the imaging plane is precisely orthogonal to the long axis of the nerve, as shown in Figures 3 and 4. Finally the paraneural fascia, the outermost connective layer that surrounds the whole fascicles within the epineurium, is hyperintense in the 3D SPGR fat suppressed images and hypointense in the TSE T1 weighted images.

## 4. Discussion

### 4.1. Anatomy and MR Appearance of the Peripheral Nerves

Peripheral nerves are composed of organized bundles of nerve fibers. Nerve fibers are made by the axons, long protrusions of nerve cell bodies located in the anterior horn of the spinal cord (motor neuron) or in the dorsal root ganglia (sensory neuron). Most axons are surrounded by myelin sheaths, made of Schwann cells. Myelinated and unmyelinated nerve fibers are grouped together in bundles called fascicles, dispersed in a loose connective tissue called endoneurium, and then enclosed in a membrane of flattened cells called perineurium. Nerve fascicles may have different sizes and their number may change along the course of the nerve [11]. They are held together in a fibrous connective tissue called epineurium, which gradually becomes thicker extending to the periphery. Within the epineurium, fat is present in variable amounts depending on the location (typically in higher amount where the nerve slides over bones or within osteofibrous tunnels) or between different nerves (e.g., the sciatic nerve contains more fat than the nerves of the upper limbs [12]).

The MR appearance of the perineurium and the epineurium was first described nearly two decades ago in early studies using MR microneurography [5–7]. The perineurium is the longest T2 component and appears bright in T2 weighted images. The tissue within the fascicles shows intermediate signal, while the internal epineurium is the shortest T2 component with low signal in any sequence with conventional TEs (10 ms or more). The high signal within the fascicles seen in T1 weighted images is mainly fat. This MR appearance may be related to the tissue properties. The epineurium is a dense matrix of thicker, longitudinally oriented collagen bundles that provides mechanical support. Because of this fibrous nature, the epineurium is a short T2 component and can be highlighted using ultrashort TE (UTE) pulse sequences [10]. The perineurium is a layered membrane made of interspaced flattened perineurial cells, basal laminas rich of proteoglycans, and thinner collagen bundles [13]; the higher amount of free water within the perineurium may contribute to the longer T2 seen in these studies [9].

### 4.2. Technical Aspects

The definition of MR microscopy (MRM) is relatively vague, and some definitions include use of spatial resolution on the order of 100 *μ*m or smaller [14]. The goal of MRM is to achieve a combination of high spatial resolution and adequate SNR with an acceptable acquisition time. In practice this is challenging, since smaller voxel size results in a decrease of SNR and a longer acquisition time. High field magnets (3T or more) and dedicated small surface coils are typically required to obtain high resolution while maintaining adequate SNR [15].

SNR increases approximately linearly with the field strength. However there are some drawbacks using high field strength such as changes in relaxation times (T1 increases, T2^*^ decreases) and increase of susceptibility effects.

Small surface coils typically have a good SNR close to the coil and allow smaller FOVs; however the sensitivity to the signal drops as the distance increases. Solenoid coils with dimensions smaller than 1 mm allow for resolutions down to the cellular scale; however this can only be used with small ex vivo samples [16]. In the clinical setting, use of the carotid 6-channel coil may limit spatial resolution, but allows for imaging of larger body regions. Furthermore, the multiple channels allow for the possibility of parallel imaging with a further reduction in scan time.

Peripheral nerves demonstrate anatomical features that are advantageous for imaging with MR microscopy. They are anisotropic in shape and they are made of fascicles oriented along one predominant direction. This feature allows for larger slice thickness to be used (e.g. 2 mm), with corresponding increases in SNR, while minimizing the detrimental effects of partial volume averaging. An important technical point is that the acquisition plane should be precisely orthogonal to the long axis of the fibers. Moreover, many clinically relevant nerves of the upper and lower limbs (including tibial, common peroneal, median, and ulnar nerves) are close to the skin surface and accessible with small surface coils.

With regard to scanning technique, early imaging parameters of peripheral nerves typically utilized fat suppression based on chemical selective saturation pulses or the STIR method [17] and fast T2 weighting techniques such as fast spin echo [18]. T2 weighted fat saturated images are useful to demonstrate the presence of edema or hyperemia. Moreover, fat saturation eliminates chemical shift artifact from the epineurial fat along the frequency encoding direction which may obscure the visualization of the perineurium [6]. However additive lengthy spatial-spectral pulses or fat-saturation pulses are required in standard fat saturation techniques, with detriment in SNR and increase in scan time. The sequence 3D IDEAL (Iterative Decomposition of water and fat with Echo Asymmetry and Least-squares estimation—GE Healthcare, USA) do not use additive fat-saturation pulses but provides a three-point water-fat separation method to achieve the maximum possible SNR performance. IDEAL method can be combined with the SPGR sequences providing in a single scan only-fat and only-water images [19]. We did not attempt to use the IDEAL technique although this is feasible and available by other vendors (DIXON—Siemens, Germany; Mdixon—Philips, The Netherlands).

## 5. Conclusion

MR microneurography provides visualization of the ultrastructure of peripheral nerves. Nearly two decades have passed since its original introduction in the literature; however a simple and repeatable protocol for in vivo use on a clinical MR scanner has not yet been published until now. In this study, we demonstrate that the constituents of peripheral nerve such as the epineurium and perineurium can be readily visualized. Although these structures are typically ignored in conventional radiological practice, they are of great importance in the clinical arena and for the determination of patient outcomes. Future studies should be performed to explore the contribution of this technique to clinical practice. Potential applications include evaluation of the nerves in compressive syndromes, stretch injuries, metabolic diseases, and tumors. Alterations of signal on T2 weighted images, usually described as generalized edema or inflammation of the entire nerve, may be referred to the appropriate compartment such as perineurium, epineurium, or fascicles. Clear visualization of chronic changes such as fascicle involution and fat and fibrous substitution may help to discern a chronic condition from early, reversible damage. Microscopic evaluation of peripheral nerves in vivo may lead to a deeper understanding of the pathogenesis of neuropathies.

Future directions such as parallel imaging with a large array of narrow coils [20, 21] may allow for the characterization of longer nerve tracts with high resolution. Evaluation of function at the microscopic scale is already a part of the standard of care for the study of the central nervous system (including DWI and DTI techniques), and the translation of these MR microscopic techniques to peripheral nerve imaging will certainly lead to better diagnosis of peripheral neuropathies.

## Figures and Tables

**Table 1:** 

	Type	TR/TE (ms)	Flip *α*	FOV (cm)	Acq matrix	*N* of slices	Slice thick (mm)	Gap (mm)	ETL	NEX	BW (kHz)	Fat sup.	Time (min)
Localizer	3D SPGR	18/8	10	14	224 × 192	140	1	0	/	1	35	Yes	2–4
Fluid sensitive HR	3D SPGR	16/6	10	5	512 × 420	10	2	0	/	5	25	Yes	10–12
T1 weighted HR	2D FSE	625/12	90	5	512 × 420	10	2	0.5	5	6	31	No	8–10
